# Theory-Based Mobile App Intervention to Promote Healthy Salt Intake Among Adults: Randomized Controlled Trial

**DOI:** 10.2196/54174

**Published:** 2025-08-20

**Authors:** Milena Sia Perin, Maria Cecília Bueno Jayme Gallani, Eduardo Augusto Fernandes Nilson, Titilayo Tatiana Agbadje, Marilia Estevam Cornélio

**Affiliations:** 1 Faculdade de Enfermagem, Universidade Estadual de Campinas - UNICAMP, 126 Tessália Vieira de Camargo Street, Cidade Universitária, Campinas, 13083-887, Brazil, 55 19-3521-8820; 2 Faculté des Sciences Infirmières, Université Laval, Quebec, QC, Canada; 3 Fundação Oswaldo Cruz, Brasilia, Brazil; 4 Universidad Autónoma de Chile, Santiago, Chile; 5 Centre Intégré Universitaire de Santé et Services Sociaux de la Capitale-Nationale - Centre de recherche en santé durable (VITAM), Quebec, QC, Canada

**Keywords:** sodium, dietary, mHealth, mobile phone, clinical trial, self-efficacy, intention, habits, nursing

## Abstract

**Background:**

Nowadays, mobile health technology has been increasingly used for treatment and prevention at all levels of health care. Associating this technology with the promotion of healthy salt consumption—both cost-effective and cost-saving public health strategies—can reduce this risk factor that contributes significantly to the increase of noncommunicable diseases worldwide.

**Objective:**

We aim to assess the usability and the efficacy of a mobile app intervention—“Sal na Medida” app—on the promotion of a healthy salt intake among adults, based on the Behavior Change Wheel framework. Additionally, to investigate if intention, self-efficacy, and habit variables mediated the effect of intervention on salt intake behavior.

**Methods:**

An experimental, randomized, and longitudinal study that evaluated the intervention effect with assessments at baseline, postintervention (1 mo), and at follow-up (2 mo). Sociodemographic and clinical data were collected from participants recruited at primary health care centers. The behavior of salt intake and per capita salt consumption were the primary outcomes. Psychosocial variables of intention, self-efficacy, and habit were assessed as possible mediators. Usability was evaluated after 1 month and 2 months of using the app.

**Results:**

Eighty-six participants were randomized in the intervention group (IG; n=43) or control group (CG; n=43). Most of the participants were female (IG: n=36, 84% and CG: n=37, 86%). Usability of app intervention scored 77.8 points (on a scale of 0 to 100) among IG participants. There was a significant reduction in salt intake in IG according to the variables of per capita salt and the behavior of salt intake. Furthermore, at the end of the follow-up, individuals in IG were 63% more likely to have a lower salt intake than those in the CG. The regression analysis showed an increase in intention and the perception of self-efficacy, and a more pronounced reduction in the habit of using more than 3 g of salt/day in preparing meals in IG when compared to CG. Habit and self-efficacy were identified as mediators of the intervention’s effect.

**Conclusions:**

The theory-based mobile app intervention for reducing salt intake has shown promise both in terms of usability and efficacy among adults. Conducting further studies to assess its potential for implementation on a larger scale would be valuable for determining its real-world impact and feasibility.

## Introduction

### Background

Recent literature indicates that excessive salt consumption worldwide is responsible for approximately 3 million deaths—more than half of all diet-related deaths—and 70 million disability-adjusted life years (DALYs), which accounts for two-thirds of DALYs [1]. In Brazil, the economic burden is particularly significant, with an estimated annual cost of US$ 102 million due to public hospitalizations [2]. These detrimental outcomes could be prevented or at least mitigated by adhering to the World Health Organization’s recommendation of a daily salt intake of 5 grams (roughly 2 grams of sodium) [3]. Additionally, the World Health Organization’s global action plan for the prevention and control of noncommunicable diseases 2013‐2020 has prioritized salt reduction as 1 of 9 voluntary global targets [4].

To reduce the problem of salt overconsumption, it is crucial to understand the different sources of salt intake, which can differ across countries and regions. In South Asia, most salt intake comes from added salt during cooking and discretionary use at the table [5]. In the Eastern Mediterranean Region, bread and dairy products are the primary sources of salt consumption [6]. In Europe and North America, processed and ultraprocessed foods contribute the most to salt intake [7]. In Brazil, table salt and salt-based spices account for approximately 74.4% of the total household sodium availability [8], with about 70% of the salt consumed coming from the addition of salt to home-cooked meals [9].

Reducing salt intake in household diets offers significant health benefits [10-12]. A 10% reduction in salt consumption could prevent 5.8 million DALYs annually due to cardiovascular disease, at a population-weighted average cost of US $1.13 per capita [13]. To achieve this reduction in salt intake, various behavioral initiatives are used, including health education interventions, public awareness campaigns, and multicomponent strategies [14].

However, interventions grounded in established theories and frameworks tend to produce more effective health outcomes, since theories allow us to identify effective intervention content and processes through which it is expected to influence behavior, and also to understand the mechanisms by which the interventions work [15]. In this way, the Behavior Change Wheel (BCW) guide [16] has been broadly used to design health interventions due to its comprehensive methodology aimed at fostering behavior change. In the field of eating behavior, the BCW framework has been successfully applied in intervention studies [17,18].

Considering the findings from previous studies on salt intake behaviors in Brazil [8,9,19], which highlighted the excessive use of salt in food preparation, the need to develop an intervention to reduce salt consumption became apparent, which led to the development of a theory-based intervention using a rigorous methodological approach, the BCW, as a guiding model, to ensure the effectiveness of the intervention.

The process of intervention design based on BCW is detailed in a previous study [20]. During the development of the intervention, it was possible to identify, by means of a literature review, that the behavior of salt addition during food preparation was the main source of the overall daily salt intake of adults and elderly Brazilians [8,9,19]. In turn, this behavior was determined by intention, self-efficacy, and habit [21]. To address these determinants and by applying the BCW steps, we were able to identify the behavior change techniques: goal setting (behavior), problem-solving, goal setting (outcome), action planning, commitment, feedback on behavior, self-monitoring of behavior, social support (unspecified), social support (practical), instruction on how to perform the behavior, information about health consequences, demonstration of the behavior, prompt or cues, behavioral practice or rehearsal, credible source, and adding objects to the environment [20].

Another critical step in the intervention development is to identify the most effective method for delivering the intervention. A review of the literature revealed the potential benefits of mobile apps and the efficacy, reliability, and association with improved health outcomes of mobile app-based interventions on eating behaviors [22-24], and specifically in interventions related to salt consumption [25]. This evidence informed our decision to use a mobile app as the delivery method for the intervention [20].

Subsequent to the intervention development process, the next step is to evaluate the effect of the intervention. Therefore, this study shows the results of the efficacy and usability assessment of a mobile phone app intervention to promote healthy salt intake behavior among adults, focusing on behavior change in adding salt during food preparation.

### Objective

This study aimed to assess the usability and the efficacy of a mobile app intervention, named “Sal na Medida” app, on the promotion of a healthy salt intake behavior among adults, based on the BCW framework. Additionally, it aimed to investigate if intention, self-efficacy, and habit serve as mediator variables of intervention effects.

## Methods

### Trial Design

This is a randomized controlled trial, with 2 parallel groups (intervention [IG] and control group [CG]). This study was conducted between October 2021 and August 2022, part of a large study developed in three modules: (1) intervention design, (2) development of “Sal na Medida” app, and (3) implementation and evaluation of the intervention efficacy and app usability. Details of the protocol of this study, with data from modules 1 and 2, have been previously described [20]. This paper adheres to the CONSORT (Consolidated Standards of Reporting Trials) guidelines for reporting randomized trials (Checklist 1) [26]. The trial was registered at The Brazilian Clinical Trials Registry (RBR-4s8qyyq) [27], date of registration: January 28, 2022.

### Participants

Participants were recruited from primary health care centers (PHCC) in a Brazilian city with a population of 51,000 people, located in the interior of São Paulo state. Inclusion criteria comprised individuals aged 20 to 59 years with adequate oral and reading skills. Participants were also required to possess sufficient digital literacy to operate the app independently or with assistance from an adult or family member. Exclusion criteria included individuals without access to a mobile phone equipped with the Android operating system. Individuals who met all the inclusion criteria and did not present any exclusion criteria were invited to participate by either the researcher or the Community Health Agent, who serves as the link between the PHCC and the families within the covered area.

### Intervention

The intervention, delivered via a mobile app, was developed using the BCW [16], as detailed in a previous study protocol [20]. The intervention development was based on the results of previous studies that identified, in the Brazilian population, a high salt intake and its sources of consumption [8,9,19] and that identified that intention, self-efficacy, and habit were the key psychosocial determinants of salt intake behavior [21].

To address these determinants and by applying the BCW framework, we selected corresponding intervention functions, that is, broad categories of things one can do to change behavior, which included education, persuasion, incentivization, modeling, enablement, and training. Building on these intervention functions, specific “active ingredients” were identified to facilitate behavioral change, referred to as behavioral change techniques. These techniques included—problem solving, goal setting (outcome), action planning, commitment, feedback on behavior, self-monitoring of behavior, social support (unspecified), social support (practical), instruction on how to perform the behavior, information about health consequences, demonstration of the behavior, prompts or cues, behavioral practice or rehearsal, credible sources, and adding objects to the environment [20].

The prototype of the mobile app, named “Sal na Medida” app, was pretested among potential users, and the final version was produced.

**Figure 1.**
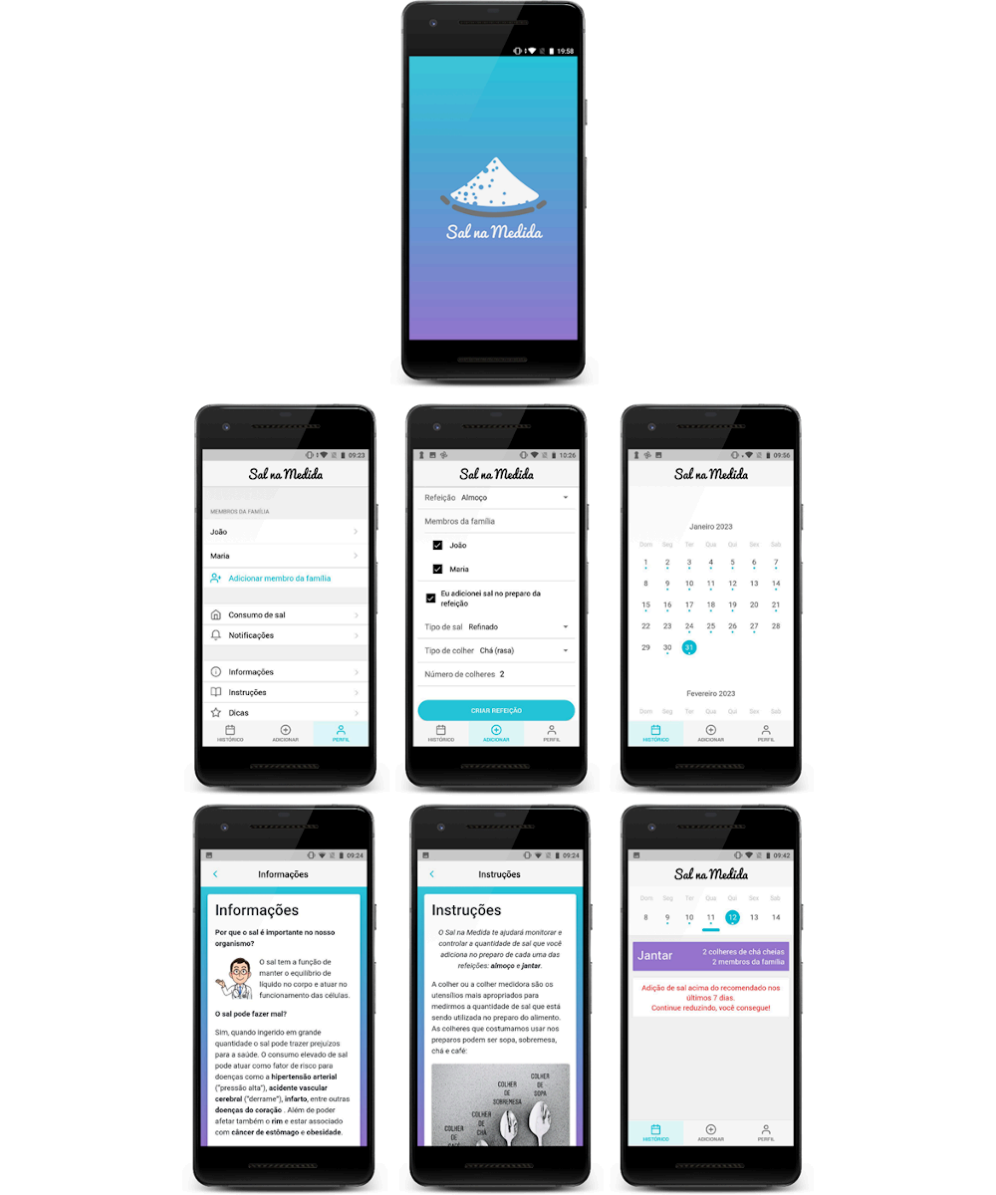
The “Sal na Medida” app screenshots.

In the app, the behavior change techniques described above were incorporated. Each day, participants record the number of people who had meals at home. For each meal, they enter the number of individuals who consumed it, as well as the number and type of salt spoons used. Additionally, the app provides information on the recommended daily salt intake, the health risks of excessive consumption, tips for reducing salt intake, and demonstrations—through text, images, and videos—to support better understanding. Throughout this process, the app offers feedback on whether the participant is meeting the recommended daily salt intake goal (Figure 1).

After the app was installed on the Android (Google LLC) phones of the participants in the IG, they received instructions on how to use it, and then they were followed up for 2 months. Participants in the CG did not receive any intervention. However, as they were under the usual care of the primary care center, they may have been exposed to general advice on salt intake that is usually given during consultations with health professionals.

### Outcomes

#### Primary Outcomes (Salt Intake Variables)

- Per capita salt: this is a self-reported questionnaire that estimates the discretionary sodium intake from salt used at the table and during home food preparation, developed and used in previous Brazilian context studies [9,19,21,28]. It measures the amount of salt consumed per person daily based on reports of the amount of salt used per month in the household (1 kg packages), taking into account the number of people living in the house and the number of meals each person eats at home.
- Behavior of salt intake: the instrument used in this study was developed in a previous study [29] as a subjective tool for collecting data on salt consumption, specifically regarding the behavior of adding salt to food. It includes a question with 5 response options, assessing the behavior of adding a maximum of 3 g of salt per day during cooking.

#### Secondary Outcomes (Psychosocial Variables)

Psychosocial variables were assessed using a validated instrument developed in a previous study [29].

- Intention: participants’ motivation for adding a maximum of 3 g of salt per day during cooking was estimated using a scale comprising 6 items, with each item rated on a 5-point scale (1-completely disagree to 5-completely agree). A high score indicated a high intention to perform the behavior.
- Self-efficacy: participants’ confidence in their ability to add a maximum of 3 g of salt per day during cooking was assessed by means of the mean of 3 items rated on a 5-point scale (1-completely disagree to 5-completely agree). The higher the score, the greater the perception of self-efficacy to perform the behavior.
- Habit: evaluation of participants’ habits regarding salt intake during cooking was conducted using a scale consisting of 10 items, each assessing the extent to which they used more than 1 level teaspoon of salt per day (equivalent to more than 3 g of salt), assessed on a 5-point scale (1-definitely not to 5-definitely yes]. Higher scores on the scale indicate a greater habit of using more than 3 g of salt per day during cooking.
- System Usability Scale (SUS): a reliable and widely used tool for assessing the usability of various products and services, including software, websites, and mobile applications [30]. It consists of a 10-item questionnaire with 5 response options for respondents (1-completely disagree to 5-completely agree). A higher score indicates better usability. The scale was applied at T_1_ and T_2_ only for IG participants.

### Data Collection

**Figure 2.**
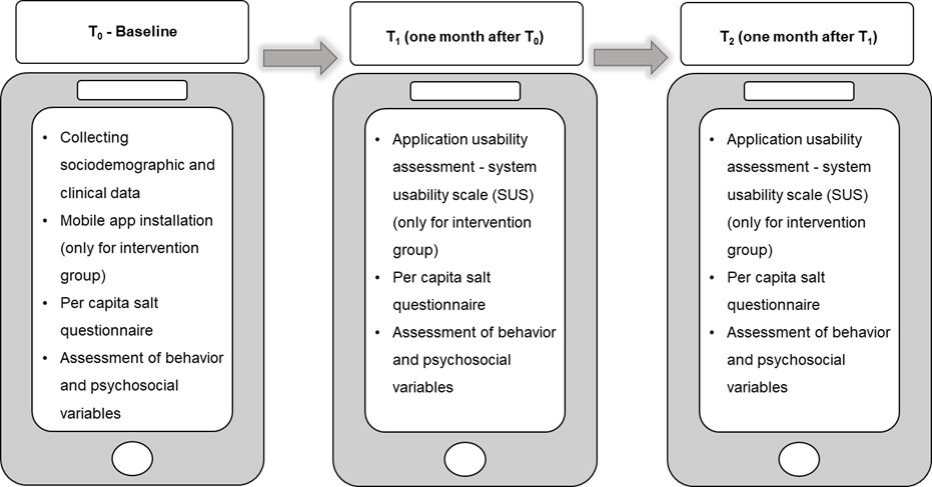
Data collection plan: control and intervention group.

This study was conducted over 3 waves of data collection during a 2-month follow-up period (Figure 2). At baseline (T_0_), sociodemographic and clinical variables were obtained from participants in both IG and CG. Variables including per capita salt, behavior of salt intake, and the psychosocial factors (intention, self-efficacy, and habit) were evaluated at T_0_, T_1_, and T_2_ for both groups. The SUS was applied to the IG at T_2_.

### Sample Size

The sample size was estimated using the G* Power software (version 3.1.9.4; Heinrich-Heine-Universität Düsseldorf), based on the objective of comparing quantitative outcomes between IG and CG, in the 3 periods of data collection. Repeated measures ANOVA was used with a significance level of 5%, 80% power, and an effect size of 0.25, which is considered a moderate degree of effect size [28]. A minimum sample of 86 participants was estimated (each group consisted of 43 participants).

### Randomization

Participants were randomly allocated to either the IG or the CG using a randomization procedure with variable block sizes. The randomization sequence was generated by a statistician, independent of the research team, using a web-based tool. The principal investigator was responsible for enrolling participants and assigning them to their respective groups according to the predefined randomization sequence and order of enrollment.

### Statistical Methods

Qualitative data were described using frequencies and percentages, and quantitative data as measures of central tendency (mean and median) and dispersion (SD, maximum, and minimum).

To evaluate the efficacy of the intervention on primary outcomes (salt intake variables), a linear regression model via generalized estimating equations modeling was used to make comparisons between groups and times.

Regression analysis with generalized linear models was used to assess the prediction of per capita salt at T_1_ and T_2_ by the psychosocial variables of habit, self-efficacy, and intention measured at T_0_.

The app’s usability was evaluated by the mean (SD) obtained by the SUS at T_2_. The SUS scores were classified as: above 90 points and the app is considered to have the best possible usability, 81 to 90 points and it is considered excellent, 71 to 80 points as good, 60 to 70 points as 0kay, and below 60 points and the degree of usability is not acceptable [31].

To assess the mediators of the effect of the intervention, mediation analysis using linear regression models was used via generalized linear models and modified Poisson regression models with robust variance. The results of the linear regression and Poisson models are presented using the estimates of the regression coefficients and relative risk, respectively, with CI values and *P* values. In the mediation analyses, the results of behavior at T_0_ were considered as a control variable.

A significance level of 5% will be adopted. Statistical analyses were performed using the SAS (version 9.4; IBM Corp) program.

### Ethical Considerations

This study was approved by the Research Ethics Committee of the university to which the researchers are connected (10937419.0.0000.5404), accredited by the National Research Ethics Commission, in compliance with Resolution 466/2012/CNS/MS. The procedures followed were in accordance with the ethical standards outlined in the Declaration of Helsinki. Written informed consent was obtained from all participants. All necessary measures were undertaken to ensure participant privacy, including the use of anonymized data, secure data storage, and strict confidentiality protocols throughout the research process. 

Data entered by IG participants into the “Sal na Medida” app was securely stored in the Firebase Realtime Database per Google’s privacy policy [32].

## Results

**Figure 3.**
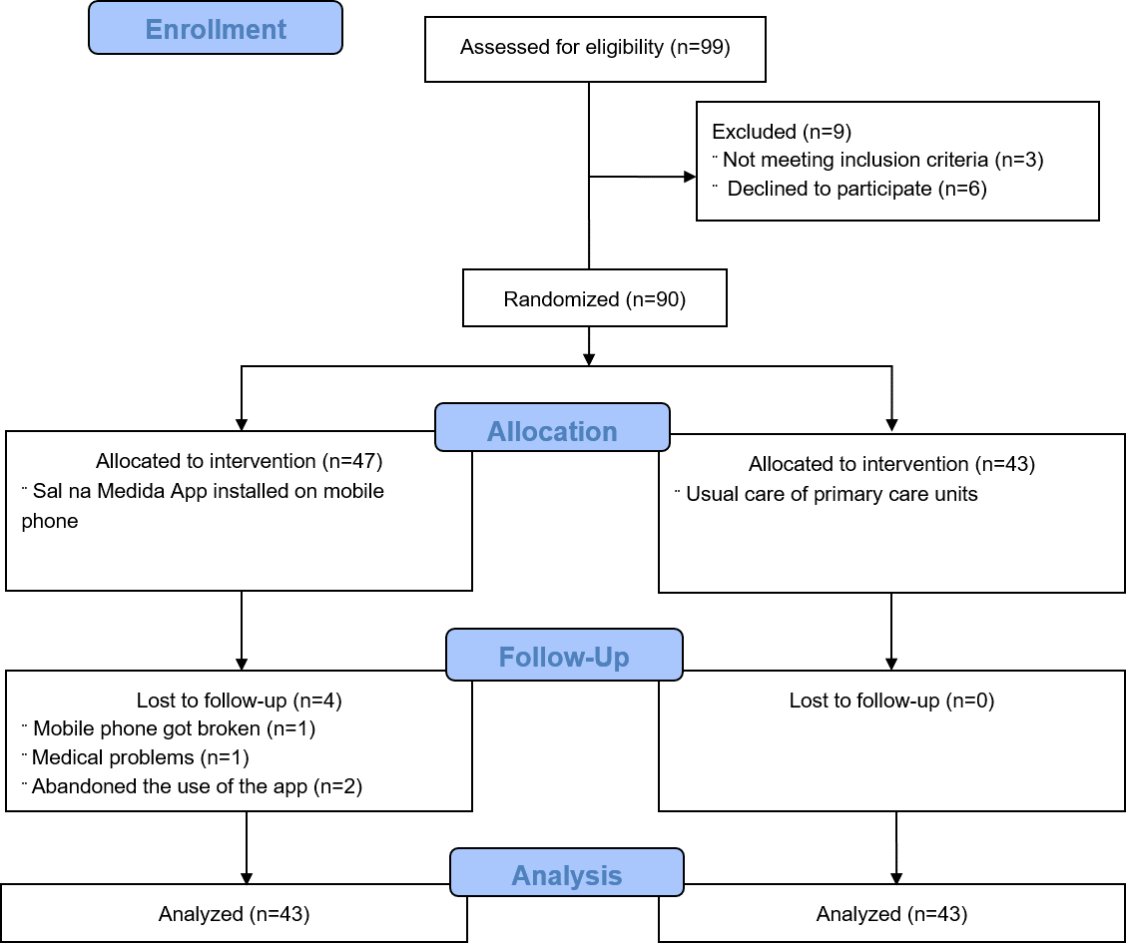
Flow diagram of the study.

A total of 90 participants were recruited, and 86 (95%) of them completed this study after 2 months of its beginning (IG n=43; CG n=43). Four participants who did not complete the study, who were predominantly female 75% (3/4), with an average age of 40.2 (SD 13.6) years and a mean of 11 (SD 2.4) years of schooling (Figure 3).

Sociodemographic and clinical characterization data, as well as comparative and association analyses applied to the 2 groups, are presented in Table 1.

**Table 1.** Sociodemographic and clinical characteristics of the intervention group and control group, and comparative and association analyses at baseline.

Variables	Intervention group (n=43)	Control group (n=43)	*P* value
Age (years), mean (SD)	34.2 (9.4)	43.4 (9.9)	<.001^a^
Schooling (years), mean (SD)	11.6 (2.9)	10.2 (1.6)	.07^b^
Individual monthly income (US $), mean (SD)	574.5 (625.8)	406.0 (185.5)	.11^b^
Family monthly income (US $), mean (SD)	1058.7 (826.6)	962.4 (757.0)	.94^b^
Skin color: White, n (%)	28 (65.1)	20 (46.5)	.08^c^
Sex: women, n (%)	36 (83.7)	37 (86)	.76^c^
Labor status: active, n (%)	40 (93)	42 (97.7)	—^d^
Hypertension	6 (13.9)	12 (27.9)	.11^c^
Diabetes	—	4 (9.3)	—
Dyslipidemia	1 (2.3)	1 (2.3)	—

a *P* value obtained with a 2-tailed unpaired Student *t* test.

b *P* value obtained with the Mann-Whitney test.

c *P* value obtained with the chi-square test.

d Not available.

Only for the age variable, there was a statistically significant difference (*P*<.001) between the means of participants in the IG (34.2, SD 9.4 y) and the CG (43.4, 9.9 y). For this reason, the analyses were adjusted according to the age variable. In both groups, there was a predominance of female (IG: n=36, 83.7%; CG: n=37, 86%), White (IG: n=28, 65.1%; CG: n=20, 46.5%), and active (IG: n=40, 93%; CG: n=42, 97.7%) participants. Considering the clinical data, there were no significant differences between groups.

The results regarding the intervention’s effect on per capita salt intake indicated that, at baseline (T_0_), there was no significant difference between the IG and CG (IG=4.6, SD 2.7 g; CG=5.3, SD 4.0 g). At T_1_, 30 days after the baseline, both groups showed a reduction in salt intake (IG=3.9, SD 2.6 g; CG=4.7, SD 3.4 g). Finally, at the end of the intervention, the IG showed a further reduction in salt consumption, while the CG remained stable (IG=3.5, SD 2.3 g; CG=4.7, SD 3.6 g; Table 2).

**Table 2.** Description of variables of salt intake (per capita salt and behavior) and psychosocial determinants (intention, self-efficacy, and habit) at T_0_, T_1_, and T_2_.

Variables	Intervention group (n=43)			Control group (n=43)		
	*T_0_*	*T_1_*	*T_2_*	*T_0_*	*T_1_*	*T_2_*
Per capita salt, mean (SD)	4.6 (2.7)	3.9 (2.6)	3.5 (2.3)	5.3 (4.0)	4.7 (3.4)	4.7 (3.6)
Intention, mean (SD)	4.0 (0.2)	4.1 (0.3)	4.2 (0.4)	3.9 (0.1)	3.9 (0.2)	3.7 (0.7)
Self-efficacy, mean (SD)	4.0 (0.2)	4.1 (0.3)	4.2 (0.5)	3.8 (0.4)	3.8 (0.5)	3.6 (0.7)
Habit, mean (SD)	3.4 (1.2)	2.3 (0.9)	1.8 (0.8)	3.2 (1.3)	2.1 (1.2)	2.6 (1.2)
Behavior (1 to 3)^a^, n (%)	24 (55.8)	7 (16.2)	3 (7)	21 (48.9)	16 (37.2)	15 (34.9)
Behavior (4 to 5)^a^, n (%)	19 (44.2)	36 (83.7)	40 (93)	22 (51.1)	27 (62.7)	28 (65.1)

a Behavior 1, 2, 3, 4, and 5 = use of a maximum of 3 g of salt/day in meal preparation—never, rarely, sometimes, on most days of the week, or every day, respectively.

In the regression analyses, the intragroup results confirmed that, at the end of the intervention, IG participants had a significant reduction in per capita salt intake (mean difference =−1.08 g; *P*=.01). In contrast, although the CG also showed a decrease in salt intake at T_2_, this difference was not statistically significant (mean difference =−0.55 g; *P*=.22). There was no statistically significant intergroup difference at T_2_ (Table 3).

**Table 3.** Effect of the intervention on the salt intake variables (per capita salt and behavior) and psychosocial variables (intention, self-efficacy, and habit) at T_0_, T_1_, and T_2_.Analyses adjusted according to the age variable.

Variables	Mean difference	95% CI		*P* value^a^
Per capita salt				
Intervention-control (T_0_)	−0.70	−2.38 to 0.98		.41
Intervention-control (T_1_)	−0.81	−2.43 to 0.80		.32
Intervention-control (T_2_)	−1.23	−2.89 to 0.43		.14
T_1_ - T_0_ (control group)	−0.58	−1.34 to 0.19		.14
T_2_ - T_0_ (control group)	−0.55	−1.43 to 0.33		.22
T_2_ - T_1_ (control group)	0.03	−0.48 to 0.53		.92
T_1_ - T_0_ (intervention group)	−1.69	−1.45 to 0.07		.07
T_2_ - T_0_ (intervention group)	−1.08	−1.91 to −0.25		.01
T_2_ - T_1_ (intervention group)	−0.39	−0.87 to 0.09		.11
Intention				
Intervention-control (T_0_)	0.17	0.07 to 0.26		<.001
Intervention-control (T_1_)	0.23	0.08 to 0.38		.002
Intervention-control (T_2_)	0.50	0.24 to 0.76		<.001
T_1_ - T_0_ (control group)	0.02	−0.07 to 0.10		.71
T_2_ - T_0_ (control group)	−0.17	−0.38 to 0.03		.09
T_2_ - T_1_ (control group)	−0.19	−0.36 to −0.02		.02
T_1_ - T_0_ (intervention group)	0.08	−0.02 to 0.18		.09
T_2_ - T_0_ (intervention group)	0.16	0.02 to 0.29		.02
T_2_ - T_1_ (intervention group)	0.08	−0.02 to 0.17		.12
Self-efficacy				
Intervention-control (T_0_)	0.22	0.01 to 0.43		.04
Intervention-control (T_1_)	0.41	0.16 to 0.66		.001
Intervention-control (T_2_)	0.66	0.35 to 0.97		<.001
T_1_ - T_0_ (control group)	−0.05	−0.22 to 0.11		.51
T_2_ - T_0_ (control group)	−0.23	−0.46 to −0.01		.049
T_2_ - T_1_ (control group)	−0.18	−0.38 to 0.03		.09
T_1_ - T_0_ (intervention group)	0.14	0.02 to 0.26		.02
T_2_ - T_0_ (intervention group)	0.21	0.03 to 0.39		.02
T_2_ - T_1_ (intervention group)	0.07	−0.08 to 0.22		.36
Habit				
Intervention-control (T_0_)	0.03	−0.56 to 0.61		.93
Intervention-control (T_1_)	−0.72	−1.22 to −0.21		.005
Intervention-control (T_2_)	−1.03	−1.52 to −0.54		<.001
T_1_ - T_0_ (control group)	−0.41	−0.68 to −0.14		.003
T_2_ - T_0_ (control group)	−0.55	−0.83 to −0.27		<.001
T_2_ - T_1_ (control group)	−0.14	−0.32 to 0.04		.12
T_1_ - T_0_ (intervention group)	−1.15	−1.48 to −0.82		<.001
T_2_ - T_0_ (intervention group)	−1.60	−1.95 to −1.25		<.001
T_2_ - T_1_ (intervention group)	−0.45	−0.62 to −0.29		<.001

a Linear regression via generalized estimating equations.

To assess the effect of the intervention on the behavior variable of salt intake, participants were considered to have achieved the desired behavior if they reported scores of “4” or “5,” indicating that they used a maximum of 3 g of salt per day in meal preparation on most days of the week or every day, respectively.

We found a significant change in the scores among IG participants. At T_0_, the majority (n=24, 55.8%) reported never, rarely, or occasionally using a maximum of 3 g of salt per day during meal preparation. By the end of the intervention (T_2_), this percentage increased significantly, with 93% reporting consistent adherence to this behavior on most or all days of the week (Table 2).

Regression intergroup analyses showed that, at T_2_, participants in the IG had a 63% higher probability of adhering to this behavior compared to those in the CG (*P*<.001; Table 3). Additionally, the intragroup analysis revealed that, after the intervention, IG participants were 111% more likely (*P*<.001) to consistently use a maximum of 3 g of salt per day when preparing meals on most days or every day (Table 4).

**Table 4.** Effect of the intervention on the salt intake variables (behavior) at T_0_, T_1_, and T_2_. Analyses adjusted according to the age variable.

Behavior	Relative risk^a^	95% CI		*P* value^b^
Intervention-control (T_0_)	0.99	0.63 to 1.56		.95
Intervention-control (T_1_)	1.52	1.15 to 2.03		.004
Intervention-control (T_2_)	1.63	1.26 to 2.11		<.001
T_1_/T_0_ (control group)	1.23	1.03 to 1.47		.02
T_2_/T_0_ (control group)	1.27	1.05 to 1.54		.01
T_2_/T_1_ (control group)	1.04	0.92 to 1.17		.56
T_1_/T_0_ (intervention group)	1.89	1.39 to 2.58		<.001
T_2_/T_0_ (intervention group)	2.11	1.52 to 2.92		<.001
T_2_/T_1_ (intervention group)	1.11	1.01 to 1.23		.04

a It was estimated the risk of presenting the result “4 and 5.”

b Poisson model modified via generalized estimating equations.

Concerning secondary outcomes, that is, the psychosocial determinants of behavior, the IG participants showed, after the intervention, a slight increase in the mean of intention (mean T_0_=4.0; mean T_2_=4.2) and in the self-efficacy (mean T_0_=4.0; mean T_2_=4.2). As for the habit of using more than 3 g of salt per day in the preparation of meals, there was a decrease in the average score of this variable in both groups, but in IG, the decrease was more pronounced (mean T_0_: 3.4; mean T_2_: 1.8) when compared to the CG (mean T_0_=3.2; mean T_2_=2.6; Table 2).

The intragroup regression analysis indicated that, at the end of the intervention, IG participants reported an increase in the intention to use a maximum of 3 g of salt per day in preparing meals (mean difference=0.16; *P*=.02). When comparing the groups, at T_2_, the IG showed higher intention when compared to CG (mean difference=0.51; *P* value<.001; Table 3).

For self-efficacy, after the intervention, there was an increase in the scores of this variable in the IG (mean difference=0.21; *P*=.02) and a decrease in the CG (−0.23; *P*=.0494). In the intergroup analysis, after the intervention, the IG presented higher self-efficacy when compared to the CG (mean difference=0.66, *P*<.001; Table 3).

Regarding the habit of using more than 3 g of salt per day in meal preparation, a significant reduction was observed in both the CG (mean difference=−0.55; *P* value <.001) and IG (mean difference=−1.60; *P*<.001). However, at T_2_, the reduction was significantly greater in the IG compared to the CG (mean difference=−1.03; *P*<.001; Table 3).

**Figure 4.**
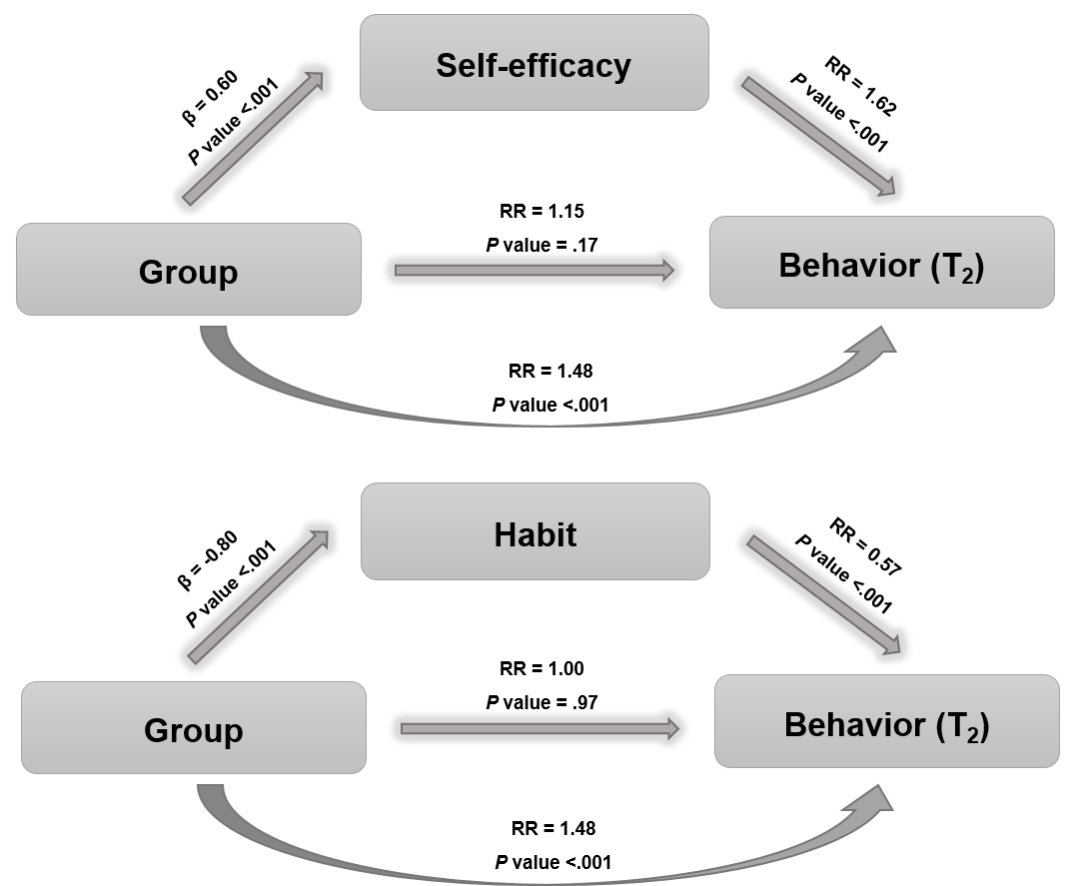
Mediation analysis. Analyzes adjusted according to the behavior T_0_. It was estimated the risk of presenting the result "4 and 5“(use of a maximum of 3 g of salt/day in the preparation of meals on most days of the week or every day, respectively). β: coefficient; RR: relative risk.

Mediation analyses found that self-efficacy and habit mediated the effect of the intervention. The analyses show that the intervention led to an increase in participants’ perception of self-efficacy, which in turn contributed to improving the behavior of salt intake, that is, the increase in self-efficacy led to a higher frequency of the behavior of using no more than 3 g of salt per day. Additionally, the intervention resulted in a decrease in the habit of adding large amounts of salt when cooking, which also served as a pathway through which the intervention influenced the behavior of salt addition (Figure 4).

The usability of the application evaluated in the IG indicated a mean score of 77.8 (SD 12.7). The average score on the SUS scale of the individuals after the intervention was 77.8, which indicates good usability of the application.

## Discussion

### Principal Findings

The “Sal na Medida” app was developed to contribute as a public policy strategy to change the behavior of adding large amounts of salt during home food preparation by adults in the general Brazilian population. Our results showed a substantial positive impact on participants’ salt intake behavior throughout the intervention and indicate that the “Sal na Medida” app is an important method for changing the behavior of salt addition in home-cooked meals and proved to be a good alternative in promoting healthy salt intake.

The improvement in the behavior among participants in the IG from baseline to the end of the intervention was quite significant. While the majority initially reported limited salt addition only occasionally or rarely at baseline, postintervention 93% reported consistently adhering to the maximum 3 g of salt per day during cooking. This highlights the efficacy of the intervention in promoting healthier salt intake behavior. Furthermore, the chances of an individual performing the healthy salt intake behavior using the mobile app were 63% higher than for people who did not use this tool.

When evaluating other review studies, we found many have been using mobile apps to change eating habits [22,23], but still, few have been in the area of salt intake specifically. As we found several sources of consumption of this nutrient, strategies were applied to reduce the consumption of high-sodium foods by offering an alternative to low-salt foods in these places where processed or ultraprocessed foods are sold or consumed [33,34]. In another study, despite adding information about using less salt when cooking or at the table, this guidance was provided by a health professional in a face-to-face visit, and the app was used only, as well as in previous studies, to encourage individuals to make choices with low sodium content in industrialized foods [35].

Another finding of our study is with respect to the mediation analyses, which revealed important insights into the mechanisms through which the intervention was successful in changing the salt intake behavior. Specifically, we found that the positive effect of the intervention on salt intake behavior was mediated by 2 psychosocial variables: self-efficacy and habit. This means that as individuals gained confidence in their ability to control their salt intake, they were more likely to adopt this behavior; additionally, breaking the automaticity in the use of salt, that is, making a conscious behavior, people are able to achieve the desired behavior. So, understanding these mediating factors clarifies the mechanism through which the intervention influences the desired results [15].

Despite providing advice and knowledge about healthy salt recommendations, interventions are generally aimed at a specific public, with the disease already triggered and with the aim of not aggravating this condition, as in cases of hypertension [34,35] or cardiovascular disease [33] or even controlling the associated symptoms, such as high blood pressure, and not the general population to prevent these diseases.

In terms of health prevention, such a tool is essential to promote public policies at the first level of health care, as actions aimed at reducing the addition of salt aim to prevent the onset and worsening of chronic noncommunicable diseases, impacting health as well as the economy [36]. Intervening in this behavior is part of several government initiatives, as this behavior of salt consumption above the recommended level is not unique to Brazil, but also to other countries in the Americas [37]. The Pan American Health Organization, together with the International Development Research Center, developed a report for Latin American countries with social marketing to help reduce salt, but it has been insufficient to implement or adopt salt reduction strategies in local communities [37].

In Brazil, there are ongoing policies, such as voluntary targets for reducing the sodium content in processed and ultraprocessed foods, the front labeling of warnings that was approved by Brazilian Health Regulatory Agency (Anvisa) and includes the alert for excess sodium [38], in addition to the recommendations of the Food Guide Brazilian, when it comes to the use of small amounts of culinary ingredients [39].

Finally, in evaluating the app usability, it is possible to identify how easily and efficiently users can interact with an app to achieve their goal. The usability score of the “Sal na Medida” app showed good usability, which is a good indication. Higher usability leads to greater user acceptance and improved health outcomes, making it a crucial factor in evaluating digital health applications [40]. Although the study involved a younger adult sample—who might generally be more tech-savvy and thus could have influenced the app’s usability score—a systematic review on educational technology usability using the SUS found no significant correlation between SUS scores and participants’ age [41]. The “Sal na Medida” app was tested with the population that uses primary health care services in Brazil, which has the potential to be another alternative tool in the future.

### Strengths and Limitations

A recent review on mobile health technologies to reduce salt makes 3 recommendations for future interventions in this area [25], in which the “Sal na Medida” app is included.

First, to create innovative and interactive mobile health technologies. Given that this study addresses a previously unexplored source of salt consumption in mobile apps—one that contributes to exceeding recommended daily intake limits—along with its demonstrated good usability and the ability to be used independently without professional assistance, the “Sal na Medida” app represents the first tool to integrate these concepts.

Second, to design interventions in primary care and associate them with salt reduction targets. Our study is aligned with specific public health recommendations for reducing salt intake, particularly by promoting behavioral changes in excessive salt use during meal preparation, and, additionally, it was conducted with adults recruited from PHCCs in a Brazilian city. Thus, the “Sal na Medida” app is promising with regard to its impact on reducing salt intake at the population level, especially in a specific geographical context. Although it was developed specifically for reducing salt consumption in food preparation, the app contains strategies that involve replacing foods with high sodium content with natural foods. This behavioral change can trigger changes in other behaviors related to salt intake, such as the consumption of processed foods.

The third and final item is to develop large-scale clinical trials with a robust design, incorporating an outcome variable (24 h urine sodium). We conducted a randomized controlled study, targeted at a specific behavior of salt intake, and the intervention was guided by the BCW [16], a robust framework. As the focus of the study was on behavior change—specifically reducing the amount of salt added during meal preparation—and on encouraging participants to adopt this behavior as a lasting habit in their daily lives, the 24-hour urine sodium was not assessed as a quantitative variable in this study.

The study also had some limitations. The system required the participants’ smartphones to have only Android because the app is not available in iOS (Apple Inc). Another limitation was the impossibility of blinding researchers and participants for data collection after the intervention, which could cause potential bias in the responses of individuals.

Another study limitation is the use of a self-reported questionnaire to estimate per capita salt intake. The literature points to the lithium-tagged salt method as the gold standard for measuring discretionary salt intake. However, studies using this method are often limited in size due to the significant burden it places on participants [42]. The self-reported per capita salt questionnaire used in this study was used in previous studies in the Brazilian context [9,19-21,43,44] because it allows us to capture the main source of salt consumption for this population. The 24-hour urinary sodium excretion is the gold standard for objectively quantifying salt intake; however, this method does not allow identification of the source of salt intake and has several limitations, such as intraindividual variability in urinary excretion due to factors like diet, hydration status, physical activity, stress, and renal function; inconvenience and burden for participants to collect the 24-hour urine sample, which may lead to incomplete collection; and high costs of analyses [45].

Finally, future studies investigating the long-term effects of the “Sal na Medida” app on maintaining behavior change are necessary, as the follow-up period in this study was limited to 2 months. Additionally, future research should include older adults to evaluate the intervention’s efficacy and the app’s usability within this population.

### Conclusions

The “Sal na Medida” app intervention demonstrated a positive impact on salt intake behavior, enhancing participants’ intention and self-efficacy perception toward reducing salt use during home-cooked meal preparation, while also decreasing the habit of adding excessive salt. Achieving a change in behavior and integrating healthy habits of salt intake into the routine of families during meals is the goal of the “Sal na Medida” app. The next phase involves scaling up the app for broader implementation.

## Supplementary material

10.2196/54174 Checklist 1 CONSORT-eHEALTH checklist (V1.6.1).

## Abbreviations

BCW: Behavior Change Wheel
CG: control group
CONSORT: Consolidated Standards of Reporting Trials
DALY: disability-adjusted life year
IDRC: International Development Research Center
IG: intervention group
PHCC: primary health care center
SUS: system usability scale
